# Evidence for under-diagnosis of childhood acute lymphoblastic leukaemia in poorer communities within Great Britain

**DOI:** 10.1038/bjc.2012.102

**Published:** 2012-04-03

**Authors:** M E Kroll, C A Stiller, S Richards, C Mitchell, L M Carpenter

**Affiliations:** 1 Childhood Cancer Research Group, Department of Paediatrics, University of Oxford, Oxford OX3 7LG, UK; 2 Cancer Epidemiology Unit, Nuffield Department of Clinical Medicine, University of Oxford, Oxford, OX3 7LF, UK; 3 Clinical Trial Service Unit, Nuffield Department of Clinical Medicine, University of Oxford, Oxford, OX3 7LF, UK; 4 Department of Paediatric Oncology and Haematology, Children’s Hospital, The John Radcliffe, Headley Way, Headington, Oxford OX3 9DU, UK; 5 Department of Public Health, University of Oxford, Oxford OX3 7LF, UK; 6 Nuffield College, University of Oxford, Oxford OX1 1NF, UK

**Keywords:** childhood leukaemia, socioeconomic status, diagnosis, infection, delayed infection, population mixing

## Abstract

**Background:**

Recorded incidence of childhood acute lymphoblastic leukaemia tends to be lower in poorer communities. A ‘pre-emptive infection hypothesis’ proposes that some children with leukaemia die from infection without diagnosis of leukaemia. Various different blood abnormalities can occur in untreated leukaemia.

**Methods:**

Logistic regression was used to compare pre-treatment blood counts among children aged 1–13 years at recruitment to national clinical trials for acute lymphoblastic leukaemia during 1980–2002 (*N*=5601), grouped by address at diagnosis within Great Britain into quintiles of the 1991 Carstairs deprivation index. Children combining severe neutropenia (risk of serious infection) with relatively normal haemoglobin and platelet counts (lack of pallor and bleeding) were postulated to be at risk of dying from infection without leukaemia being suspected. A deficit of these children among diagnosed patients from poorer communities was predicted.

**Results:**

As predicted, there was a deficit of children at risk of non-diagnosis (two-sided *P*_trend_=0.004; *N*=2009), and an excess of children with pallor (*P*_trend_=0.045; *N*=5535) and bleeding (*P*_trend_=0.036; *N*=5541), among cases from poorer communities.

**Conclusion:**

Under-diagnosis in poorer communities may have contributed to socioeconomic variation in recorded childhood acute lymphoblastic leukaemia incidence within Great Britain, and elsewhere. Implications for clinical practice and epidemiological studies should be considered.

Record-based studies have reported higher childhood leukaemia incidence rates in relatively affluent communities within many different countries (Borugian *et al*, 2005; Poole *et al*, 2006; Adelman *et al*, 2007), and internationally (Feltbower *et al*, 2004). Within England and Wales, the leukaemia rate for the most deprived fifth of the child population was consistently about 90% of the rate for the most affluent fifth, in each of the decades centred on the census years 1981, 1991, and 2001 (Kroll *et al*, 2011b). The apparent socioeconomic gradient occurs both for childhood leukaemia as a whole, and for the lymphoid subtype (∼80% of childhood leukaemia in developed countries), which in children consists almost entirely of acute lymphoblastic leukaemia. The socioeconomic gradient might be related to either of the two well-known aetiological hypotheses linking risks of childhood leukaemia with unusual patterns of infection: ‘delayed infection’ (Greaves, 2006) and ‘population mixing’ (Kinlen, 2011). However, under-diagnosis of leukaemia in poorer children, as proposed by a ‘pre-emptive infection hypothesis’ (Stewart, 1961; Greaves *et al*, 1985; Doll, 1989), is another possible explanation.

Acute leukaemia in children is not always easy to diagnose, as the clinical signs can be ‘vague and non-specific’ (Mitchell *et al*, 2009a). Neutropenia frequently occurs in untreated leukaemia, and predisposes to bacterial and fungal infections, which can lead to death from pneumonia, septicaemia, or meningitis (Baehner, 1996). Bone marrow examination is necessary for definitive diagnosis of leukaemia, but is not routinely performed in cases of severe infection (Gillespie *et al*, 2004). As neutropenia is a well-recognised sign of severe infection, some children suffering from leukaemia might die from infection without leukaemia ever being suspected. Within Great Britain, this might have happened more frequently in poorer communities, where primary health care provision has been less generous and childhood infection mortality rates have been higher (Reading, 1997; Health Protection Agency, 2005). If so, the recorded incidence of childhood leukaemia would be lower in poorer communities. For acute lymphoblastic leukaemia, the effect might be clearer in B-precursor than T-precursor disease, because obvious enlargement of the lymph nodes is less frequent in B-precursor cases (Greaves *et al*, 1985).

Untreated acute lymphoblastic leukaemia can cause various different blood abnormalities, in almost any combination. Unlike neutropenia, low levels of haemoglobin and platelets produce obvious clinical signs that strongly suggest leukaemia: pallor and bleeding, respectively. We postulated that children who combined severe neutropenia with relatively normal haemoglobin and platelet counts would be at risk of dying from infection without leukaemia being suspected, and predicted that these children would be under-represented among poorer patients.

## Materials and methods

Using records from four consecutive clinical trials sponsored by the Medical Research Council (Eden *et al*, 2000; Mitchell *et al*, 2009b), pre-treatment haemoglobin, platelet, and (to September 1990) neutrophil counts were obtained for children who had been diagnosed with acute lymphoblastic leukaemia in the United Kingdom during September 1980 to November 2002. The study was restricted to the trial participants who were resident in Great Britain and diagnosed between the first and the fourteenth birthday (ages 1–13 years), as trial participation rates were relatively low at ages <1 and 14 years, and addresses were not available for older recruits, or those resident in Northern Ireland.

Address at diagnosis was obtained by linking the trial data to the National Registry of Childhood Tumours, which records cancer diagnosed since 1962 in residents of Great Britain under 15 years old (Kroll *et al*, 2011a). Cases were grouped according to the deprivation category of the 1991 census ward containing the postcode of the address at diagnosis (1=affluent, … 5=deprived; Office for National Statistics, 2010), using national child-population-weighted quintiles of the 1991 Carstairs deprivation index (Carstairs and Morris, 1989). Cases were classified by immunophenotype, and the T-precursor and unknown subgroups were combined for analysis.

Using the pre-treatment blood count, each patient was classified as being with or without risk of pallor, of bleeding, and of sepsis, when diagnosed with leukaemia. Following conventional clinical criteria, risks of bleeding, and sepsis were defined, respectively, as platelets <20 × 10^9^ l^−1^ (Gaydos *et al*, 1962) and neutrophils <0.5 × 10^9 ^l^−1^ (Bodey *et al*, 1966); risk of pallor (very severe anaemia) was defined as haemoglobin <5 g dl^−1^. Cases at risk of non-diagnosis were defined as those at risk of sepsis without pallor or bleeding (i.e., the combination of neutrophils <0.5 × 10^9^ l^−1^, platelets ⩾20 × 10^9 ^l^−1^, and haemoglobin ⩾5 g dl^−1^).

Logistic regression was used to estimate associations between deprivation (in quintile categories) and the odds of having been at risk of pallor, bleeding, sepsis, or non-diagnosis (each treated as a dichotomous variable), in successive univariate analyses. Terms representing effects of individual trial periods, and their interactions with deprivation, were included in preliminary models, but dropped because they were not statistically significant. Goodness-of-fit was assessed by Pearson’s *χ*^2^ test and found acceptable. Models were compared by the likelihood ratio test. All statistical tests were two-sided, using the significance level of 5%.

## Results

After excluding 27 registered cases with incomplete address information, 5601 cases were eligible for analysis. These comprised 79% of children aged 1–13 years who were resident in Great Britain and diagnosed with acute lymphoblastic leukaemia during the study period. Pre-treatment haemoglobin and platelet counts were recorded in all four trials, whereas neutrophil counts were recorded in the two earlier trials only. Hence, classification according to risk of non-diagnosis of leukaemia was possible for 2009 cases. The distributions of cases by level of deprivation, age at diagnosis, cell type, and available blood counts were similar in all trials (Table 1).

As predicted, there was a deficit of children at risk of non-diagnosis among cases from more deprived communities (Table 2). The odds ratio for risk of non-diagnosis per quintile of deprivation was 0.90 (95% confidence interval 0.84–0.97; *P*_trend_=0.004; *N*=2009); comparing the most deprived fifth of the population with the most affluent fifth, the odds ratio was 0.68 (0.48–0.96). The patterns observed separately for pallor (*P*=0.045; *N*=5535), bleeding (*P*=0.036; *N*=5541), and sepsis (*P*=0.083; *N*=2027) were weaker, but each trend went in the direction expected: odds of pallor and bleeding increased with deprivation, whereas odds of sepsis decreased. Similar trends were evident for B-precursor cases separately (*P*_trend_=0.002; *N*=1728) but not for other/unspecified cases (*P*_trend_=0.894; *N*=281). Restricting the analyses for pallor and bleeding to the earlier trials reduced the statistical significance but did not change the directions of the associations (not shown).

## Discussion

For acute lymphoblastic leukaemia, these findings are consistent with the ‘pre-emptive infection hypothesis’, which proposes that some children with leukaemia die from infection without leukaemia being suspected. Among diagnosed cases from poorer communities, there was an excess of children with blood counts implying obvious clinical signs of leukaemia (pallor and bleeding) and a deficit of children with blood counts implying risk of dying from infection without diagnosis of leukaemia (sepsis without pallor or bleeding). As predicted, similar results were obtained for the B-precursor subgroup alone, but not for the other/unspecified subgroup consisting mainly of T-precursor cases, in which obvious enlargement of the lymph nodes is typically more frequent.

The results must be interpreted with caution. As the study was restricted to children with acute lymphoblastic leukaemia, the findings cannot be generalised to those with myeloid or mature lymphoid leukaemia. The main results are limited to the two clinical trials that recruited patients during 1980–1990, because neutrophil counts were not available for patients enrolled in the later trials: consistent results were, however, obtained for clinical signs in patients from all four trials up to 2002. The blood count data were derived from paper forms that may not always have been filled in correctly, and there may have been inaccuracies in the immunophenotype data, particularly for cases in the earlier two trials: nevertheless, there is no reason to suspect systematic error in the blood counts, and the associations were evident when all cases were included, regardless of immunophenotype. Finally, the diagnostic process includes safeguards, and blasts should have been recognised in the peripheral blood, or bone marrow abnormalities detected at autopsy – but, by definition, there would be no record of any cases that were missed.

Under-diagnosis may have contributed to the decreased leukaemia incidence in poorer children that has been reported within many different countries, and internationally. Other explanations might include rate calculation artefacts, registration bias, or a real increase in risk caused by some factor associated with higher socioeconomic status. For the cited comparison within England and Wales, artefact caused by numerator/denominator discrepancy seems unlikely, as rates were calculated from registry data for three separate decades, each centred on a census year, using appropriate census populations and census-specific deprivation indices (Kroll *et al*, 2011b); and a detailed study of cases diagnosed during 2003–2004 found very little evidence of socioeconomic variation in completeness of registration (Kroll *et al*, 2011a). The ‘delayed infection’ (Greaves, 2006) and ‘population mixing’ (Kinlen, 2011) hypotheses both propose that exposure to infection triggers leukaemia in susceptible children; in particular, the ‘delayed infection’ hypothesis suggests that protection from infection in infancy is a predisposing factor for common (B-precursor) acute lymphoblastic leukaemia. Either or both of these hypotheses could explain a reduction in childhood leukaemia rates in poorer communities, on the assumption of greater exposure of infants to infection and/or reduced population mobility in poorer communities (Dockerty *et al*, 2001; Stiller *et al*, 2008). Conversely, under-diagnosis caused by ‘pre-emptive infection’ in poorer communities might explain some of the existing epidemiological evidence for associations of higher childhood leukaemia risk with delayed infection, population mixing, or other factors linked to higher socioeconomic status. However, no explanation other than ‘pre-emptive infection’ seems likely to account for the specific patterns of blood abnormality observed in this study.

If confirmed with recent data, the findings would be relevant to future clinical practice. The apparent stability of the results over time is consistent with the persistence of the socioeconomic differential in England and Wales up to 1996–2005. However, the data relating to sepsis were available only for a limited time period (1980–1990), and the whole study period was relatively short (1980–2002); the analysis cannot be extended to more recent years, as the relevant national clinical trials have not collected pre-treatment blood counts in detail since 2002. Nevertheless, other approaches are possible. For example, previous studies from the United Kingdom have documented the presentation of childhood cancer in primary care by interviewing parents (Dixon-Woods *et al*, 2001), or examining the General Practice Research Database (Dommett *et al*, 2012); either of these sources, or Hospital Episode Statistics, might be used to investigate variation in response to signs of childhood leukaemia.

In conclusion, these results support the suggestion that childhood acute lymphoblastic leukaemia may have been under-diagnosed in poorer communities within Great Britain, and elsewhere. Potential implications for epidemiological studies and clinical practice should be considered.

## Figures and Tables

**Table 1:** The distribution of children by deprivation quintile, age group, immunophenotype, and pre-treatment blood counts, within each clinical trial period and overall

**Clinical trial**	**UKALL VIII**	**UKALL X**	**UKALL XI**	**ALL 97/99**	**All trials**
Start	September 1980	January 1985	October 1990	March 1997	September 1980
End	December 1984	September 1990	February 1997	November 2002	November 2002
Number of children	703	1395	1851	1652	5601
					
*Deprivation quintile*					
1 (affluent)	149 (21%)	270 (19%)	390 (21%)	358 (22%)	1167 (21%)
2	149 (21%)	295 (21%)	402 (22%)	329 (20%)	1175 (21%)
3	142 (20%)	277 (20%)	365 (20%)	330 (20%)	1114 (20%)
4	141 (20%)	280 (20%)	365 (20%)	347 (21%)	1133 (20%)
5 (deprived)	122 (17%)	273 (20%)	329 (18%)	288 (17%)	1012 (18%)
					
*Age at diagnosis (years)*					
1–4	387 (55%)	823 (59%)	1079 (58%)	909 (55%)	3198 (57%)
5–9	202 (29%)	406 (29%)	533 (29%)	506 (31%)	1647 (29%)
10–13	114 (16%)	166 (12%)	239 (13%)	237 (14%)	756 (13%)
					
*Immunophenotype*					
B-precursor	561 (90%)	1227 (91%)	1486 (89%)	1456 (90%)	4730 (90%)
T-precursor	61 (10%)	116 (9%)	193 (11%)	168 (10%)	538 (10%)
Unknown	81	52	172	28	333
					
*Risk of pallor*					
No (haemoglobin ⩾5 g dl^−1^)	551 (80%)	1060 (77%)	1396 (76%)	1295 (79%)	4302 (78%)
Yes (haemoglobin<5 g dl^−1^)	140 (20%)	318 (23%)	434 (24%)	341 (21%)	1233 (22%)
Unknown	12	17	21	16	66
					
*Risk of bleeding*					
No (platelets ⩾20 × 10^9^ l^−1^)	541 (78%)	1090 (79%)	1391 (76%)	1227 (75%)	4249 (77%)
Yes (platelets<20 × 10^9^ l^−1^)	157 (22%)	286 (21%)	439 (24%)	410 (25%)	1292 (23%)
Unknown	5	19	21	15	60
					
*Risk of sepsis*					
No (neutrophils ⩾0.5 × 10^9^ l^−1^)	358 (52%)	749 (56%)	—	—	1107 (55%)
Yes (neutrophils<0.5 × 10^9^ l^−1^)	324 (48%)	596 (44%)	—	—	920 (45%)
Unknown	21	50	1851	1652	3574
					
*Risk of non-diagnosis* a					
No	492 (73%)	1027 (77%)	—	—	1519 (76%)
Yes^b^	179 (27%)	311 (23%)	—	—	490 (24%)
Unknown	32	57	1851	1652	3592

a Sepsis without pallor or bleeding.

b Neutrophils <0.5 × 10^9^ l^−1^, haemoglobin ⩾5 g dl^−1^, and platelets ⩾20 × 10^9^ l^−1^. United Kingdom Medical Research Council clinical trial participants, resident in Great Britain, aged 1–13 years at diagnosis of acute lymphoblastic leukaemia 1980–2002.

**Table 2:** Odds ratio (95% confidence interval) for pre-treatment risk of pallor, bleeding, sepsis, and non-diagnosis, among children diagnosed with acute lymphoblastic leukaemia, for each quintile of deprivation relative to the most affluent, by immunophenotype and overall

**Pre-treatment risk**	**Pallor**	**Bleeding**	**Sepsis**	**Non-diagnosis**
Definition	Haemoglobin <5 g dl^−1^	Platelets <20 × 10^9^ l^−1^	Neutrophils <0.5 × 10^9^ l^−1^	Sepsis without pallor or bleeding^a^
Deprivation quintile				
				
*B-precursor*				
Number of children	4695	4700	1740	1728
1 (affluent)	1.00	1.00	1.00	1.00
2	0.91 (0.73–1.13)	1.07 (0.87–1.32)	0.94 (0.70–1.26)	1.06 (0.77–1.47)
3	1.02 (0.83–1.26)	1.12 (0.91–1.38)	1.18 (0.88–1.59)	1.22 (0.88–1.69)
4	1.02 (0.83–1.26)	1.04 (0.84–1.28)	0.85 (0.63–1.15)	0.75 (0.53–1.06)
5 (deprived)	1.24 (1.00–1.53)	1.27 (1.03–1.57)	0.75 (0.56–1.02)	0.61 (0.42–0.89)
*P*(trend)	0.027	0.064	0.059	0.002
Odds ratio per quintile	1.06 (1.01–1.11)	1.05 (1.00–1.10)	0.94 (0.88–1.00)	0.88 (0.82–0.96)
				
*T-precursor/unknown*				
Number of children	840	841	287	281
1 (affluent)	1.00	1.00	1.00	1.00
2	0.98 (0.56–1.72)	0.73 (0.38–1.41)	0.98 (0.45–2.11)	1.15 (0.47–2.84)
3	0.70 (0.38–1.30)	1.47 (0.82–2.67)	0.93 (0.42–2.06)	0.90 (0.35–2.33)
4	0.83 (0.47–1.48)	0.86 (0.46–1.62)	0.59 (0.25–1.39)	0.73 (0.26–2.02)
5 (deprived)	0.98 (0.54–1.76)	1.29 (0.70–2.40)	1.31 (0.59–2.92)	1.33 (0.52–3.40)
*P*(trend)	0.683	0.385	0.914	0.894
Odds ratio per quintile	0.97 (0.85–1.11)	1.06 (0.92–1.23)	1.01 (0.84–1.21)	1.01 (0.82–1.25)
				
*All cases*				
Number of children	5535	5541	2027	2009
1 (affluent)	1.00	1.00	1.00	1.00
2	0.92 (0.76–1.13)	1.05 (0.86–1.27)	0.93 (0.71–1.22)	1.06 (0.78–1.44)
3	0.99 (0.81–1.21)	1.17 (0.96–1.42)	1.12 (0.85–1.48)	1.16 (0.85–1.58)
4	1.00 (0.82–1.22)	1.02 (0.83–1.24)	0.82 (0.62–1.08)	0.74 (0.54–1.04)
5 (deprived)	1.22 (1.00–1.48)	1.29 (1.06–1.57)	0.80 (0.60–1.06)	0.68 (0.48–0.96)
*P*(trend)	0.045	0.036	0.083	0.004
Odds ratio per quintile	1.05 (1.00–1.10)	1.05 (1.00–1.10)	0.95 (0.89–1.01)	0.90 (0.84–0.97)

a Neutrophils <0.5 × 10^9^ l^−1^, haemoglobin ⩾5 g dl^−1^, and platelets ⩾20 × 10^9^ l^−1^. United Kingdom Medical Research Council clinical trial participants, resident in Great Britain, aged 1–13 years at diagnosis of acute lymphoblastic leukaemia 1980–2002.
